# Comparative effects of acupuncture and metformin on insulin sensitivity in overweight/obese and lean women with polycystic ovary syndrome and insulin resistance: a *post hoc* analysis of a randomized trial

**DOI:** 10.3389/fmed.2023.1232127

**Published:** 2023-12-14

**Authors:** Jie Cao, GuanHua Nie, Zhihao Dai, Dan Shan, Zhihong Wei

**Affiliations:** ^1^Department of Obstetrics and Gynecology, Jiangsu University Affiliated Peoples Hospital, Zhenjiang, China; ^2^Department of Neurology, Neuroscience Center, The First Hospital of Jilin University, Changchun, China; ^3^School of Medicine, Royal College of Surgeons in Ireland, University of Medicine and Health Sciences, Dublin, Ireland; ^4^School of Medicine, National University of Ireland, Galway, Ireland; ^5^Xijing Hospital, The Fourth Military Medical University, Xi'an, Shanxi, China

**Keywords:** acupuncture, body mass index, polycystic ovary syndrome, insulin resistance, insulin sensitivity, metformin

## Abstract

**Objective:**

This study explored the efficacy of acupuncture and metformin in enhancing insulin sensitivity among women with polycystic ovary syndrome (PCOS) and insulin resistance (IR), distinguishing between overweight/obese and lean groups.

**Methods:**

A post-hoc analysis of a randomized trial (NCT02491333) was undertaken. Participants were women aged 18–40 with PCOS and IR. They were randomized to receive true acupuncture with a placebo, metformin with sham acupuncture, or sham acupuncture with a placebo for 4 months, with follow-up visits over 3 months. Our study, involving 339 women, assessed the differential impact of acupuncture and metformin on insulin sensitivity in overweight/obese [body mass index (BMI) ≥ 24] versus lean women (BMI < 24). Primary outcomes measured changes in the Homeostatic Model Assessment of Insulin Resistance (HOMA-IR) at 4 and 7 months. Secondary outcomes assessed changes in glucose area under the curve (glucose_AUC_) during the oral glucose tolerance test (OGTT) and BMI changes at 4 months.

**Results:**

Overweight/obese participants were generally older with higher measurements in various health metrics, but lower levels in specific hormonal metrics compared to lean women (*p* < 0.05). Among overweight/obese women, metformin outperformed acupuncture in reducing HOMA-IR levels (*p* = 0.004) and showed a significant drop from the baseline after 4 months (*p* < 0.05). In contrast, acupuncture’s effect on HOMA-IR did not significantly differ from sham acupuncture at 4 and 7 months. For lean women, metformin and acupuncture showed comparable improvements in HOMA-IR, with notable declines at 4 and 7 months (*p* < 0.05). Acupuncture proved more beneficial for lean women than their overweight/obese counterparts after 4 months (*p* = 0.021).

**Conclusion:**

In overweight/obese women with PCOS and IR, acupuncture was less effective than metformin in enhancing insulin sensitivity. However, in lean women, acupuncture’s efficacy was comparable to metformin. Further studies are required to validate these observations.

## Introduction

Polycystic ovary syndrome (PCOS) significantly impacts women in their reproductive years (1). This condition is marked by chronic anovulation and hyperandrogenism. Alarmingly, nearly 40% of women with PCOS also suffer from insulin resistance (IR) (2). Obesity intensifies both the reproductive and metabolic challenges tied to PCOS. Between 35 and 60% of obese women with PCOS display a connection between the pathological mechanisms of IR and PCOS (3, 4). Additionally, weight gain can heighten IR (5). Research indicates that weight loss can effectively diminish androgen levels in PCOS patients while enhancing glucose and lipid metabolism (6). Standard PCOS treatments often involve the insulin sensitizer, metformin, coupled with lifestyle modifications (7). This strategy seeks to decrease androgen concentrations and bolster insulin sensitivity.

In recent times, acupuncture, an integral part of traditional Chinese medicine, has emerged as a promising and safe therapeutic alternative for PCOS (8). Acupuncture seems to regulate the body by stimulating sensory nerve fibers, which in turn adjust sympathetic activity in the ovaries and central nervous system (9, 10). A thorough review revealed that acupuncture may ameliorate various metabolic disorders leading to IR, such as hyperglycemia, obesity, hyperphagia, hyperlipidemia, inflammation, and impaired sympathetic nervous activity (11). Some research also suggests acupuncture can enhance insulin sensitivity alongside antidiabetic medications (12, 13).

While past research indicates that acupuncture might be less potent than metformin in enhancing insulin sensitivity, it appears superior in optimizing glucose metabolism in women with PCOS and IR (14). Notably, data comparing the efficacy of acupuncture and metformin among overweight/obese versus lean women with PCOS and IR remains scarce. Our study offers a post-hoc analysis from a randomized controlled trial, aiming to assess the effectiveness of acupuncture and metformin in boosting insulin sensitivity in women with PCOS and IR, categorized by distinct body mass index (BMI) groups.

## Methods

### Study design

The study design and subject eligibility criteria have been delineated in a prior publication (NCT02491333) (14). This research compared the effectiveness of true/sham acupuncture and metformin/placebo in enhancing insulin sensitivity among women with PCOS and IR. Out of the original participants, 339 were considered for this *post hoc* analysis, excluding 3 due to unrecorded baseline BMI data.

### Participants

As previously described, eligible participants were women aged 18–40 with a BMI >18.5 kg/m^2^, diagnosed with PCOS and IR, and with a HOMA-IR ≥ 2.14. Participants were randomized to receive either genuine acupuncture paired with placebo, metformin alongside sham acupuncture (0.5 g thrice daily), or sham acupuncture with placebo over 4 months, followed by three monthly follow-up sessions. Acupuncture (both true and sham) was administered three times weekly, with metformin or placebo dosage set at 0.5 g three times daily. Based on BMI guidelines for a Chinese cohort, individuals with BMI < 24 were labeled as lean, while those with BMI ≥ 24 were categorized as overweight/obese (15). PCOS was defined per the revised 2004 Rotterdam criteria (16), necessitating at least two of these symptoms: oligomenorrhea/amenorrhea, biochemical/clinical hyperandrogenism, or polycystic ovary morphology (PCOM). The HOMA-IR formula is given by: fasting blood glucose (FPG) (mmol/L) × fasting insulin (FINS) (μU/mL)/22.5, where a score of ≥2.14 indicates IR (17). Participants were those not seeking immediate pregnancy and committed to barrier contraception for 7 months. Exclusions encompassed women with disorders such as hyperprolactinemia, FSH >15 mIU/mL, hypothyroidism, diabetes, Cushing’s syndrome, certain cancers, and others.

### Outcomes

The primary outcome was the alteration in HOMA-IR levels between the baseline, 4-month, and 7-month marks. Secondary outcomes revolved around changes from the baseline to the 4-month point in aspects including anthropometry [BMI, waist-to-hip ratio (WHR), Acne (Rosenfield score), and hirsutism (determined by Ferriman–Gallwey score)], metabolic profiles (FPG, FINS, glucoseAUC, insulinAUC, HOMA-β, C-peptide, and hemoglobin A1c), and hormonal profiles [LH to FSH ratio (LH/FSH), total testosterone, and free androgen index (FIA)].

### Statistical analysis

Participants were grouped by their initial BMI. Continuous variables are either denoted as mean ± SD or as medians with interquartile ranges. To compare continuous variables, either the ANOVA test (for normal distributions) or the Kruskal-Wallis H test (for non-normal distributions) was employed. The Student’s *t*-test facilitated two-group comparisons, while the ANOVA was for three-group contrasts. Both univariable and multivariable binary logistic regressions ascertained the risk factors affecting HOMA-IR improvement after 4 months of treatment. A *p* < 0.05 was deemed statistically significant. Calculations utilized the IBM SPSS Statistics 25 and STATA MP 14.0 software.^1^

## Results

### Participant flow

A total of 339 women were enrolled in the *post hoc* analysis, 110 (32.45%) were lean and 229 (67.55%) were overweight/obese. Overweight/obese women who participated in the study were generally older and had higher levels of Waist, WHR, Acne score, baseline HOMA-IR, FPG, FINS, Glucose_AUC_, Insulin_AUC_, HOMA-β, C-peptide, HbA1C, TG, low-density lipoprotein cholesterol (LDL-C), and FAI, but had lower levels of baseline HDL-C, ApoA-1, Apo B, LH, FSH, LH/FSH, Total T, and SHBG (Table 1, *p* < 0.05). Among the 110 lean women, 37 (33.63%) were placed into the true acupuncture + placebo group, 36 (32.75%) were placed into the sham acupuncture + metformin group, and 37 (33.63%) were placed into the sham acupuncture + placebo group. They shared similar baseline characteristics (Table 2, *p* > 0.05). Among the 229 overweight/obese women, 75 (32.75%) were placed into the true acupuncture + placebo group, 77 (33.63%) were placed into the sham acupuncture + metformin group, and 77 (33.63%) were placed into the sham acupuncture + placebo group. They also shared similar baseline characteristics (Table 2, *p* > 0.05).

**Table 1:** Baseline clinical characteristics of the study population.

	Lean (*n* = 110)	Overweight/obese (*n* = 229)	*p*-value
Age, years	26.33 ± 4.14	27.93 ± 4.07	0.001
BMI, kg/m^2^	21.26 ± 1.76	28.69 ± 3.62	<0.001
Waist, cm	75.15 ± 6.12	93.22 ± 9.12	<0.001
WHR	0.83 ± 0.06	0.90 ± 0.06	<0.001
Acne score	0.44 ± 0.63	0.64 ± 0.86	0.012
Hirsutism score	3.34 ± 3.22	4.00 ± 4.09	0.108
Fasting serum levels			
HOMA-IR	3.18 ± 1.24	4.99 ± 2.49	<0.001
FPG, mmol/L	5.15 ± 0.37	5.34 ± 0.65	0.001
FINS, mU/L	13.87 ± 5.09	20.87 ± 9.59	<0.001
Glucose_AUC_, mmol/L × min	14.54 ± 2.88	15.82 ± 3.25	0.001
Insulin_AUC_, mU/L × min	211.04 ± 133.79	277.68 ± 148.19	<0.001
HOMA-β, %	178.57 ± 84.01	249.67 ± 144.92	<0.001
C-peptide, nmol/L	0.80 ± 0.29	1.09 ± 0.45	<0.001
HbA1C, %	5.18 ± 0.37	5.40 ± 0.38	<0.001
TC, mmol/ L	4.84 ± 0.81	5.01 ± 3.21	0.598
TG, mmol/ L	1.09 ± 0.52	1.74 ± 1.13	<0.001
LDL-C, mmol/ L	2.90 ± 0.59	3.17 ± 0.78	0.001
HDL-C, mmol/ L	1.48 ± 0.32	1.21 ± 0.27	<0.001
ApoA-1, mmol/L	1.30 ± 0.17	1.18 ± 0.19	<0.001
Apo B, mmol/L	0.83 ± 0.18	0.96 ± 0.30	<0.001
LH, IU/L	12.22 ± 6.47	9.84 ± 6.01	0.002
FSH, IU/L2	5.94 ± 1.61	5.86 ± 1.67	0.702
LH/FSH, IU/L	2.14 ± 1.10	1.71 ± 0.83	<0.001
Total T, nmol/L	2.48 ± 0.99	2.01 ± 0.96	0.001
SHBG, nmol/L	44.47 ± 20.19	28.60 ± 17.10	<0.001
FAI	6.66 ± 3.79	9.85 ± 7.18	<0.001

BMI, body mass index; WHR, waist-to-hip ratio; HOMA-IR, homeostatic model of assessment for insulin resistance; FPG, fasting plasma glucose; FINS, fasting insulin; Glucose_AUC_, the area under the curve during the oral glucose tolerance test (OGTT) for glucose (using the trapezoidal rule); Insulin_AUC_, the area under the curve during the OGTT for insulin (using the trapezoidal rule); HOMA-β, homeostatic model assessment for beta cell function; HbA1C, hemoglobin A1c; TC, total cholesterol; TG, triglycerides; LDL-C, low-density lipoprotein cholesterol; HDL-C, high-density lipoprotein cholesterol; ApoA-1, apolipoprotein A-1; Apo B, apolipoprotein B; LH/FSH, LH to FSH ratio; Total T, total testosterone; SHBG sex hormone binding globulin; FAI, free androgen index.

**Table 2:** Baseline characteristics of lean and overweight/obese groups under different treatments.

	Lean (*n* = 110)			*p*-value	Overweight/obese (*n* = 229)			*p*-value
	True acupuncture + Placebo (*n* = 37)	Sham acupuncture + Metformin (*n* = 36)	Sham acupuncture + Placebo (*n* = 37)		True acupuncture + Placebo (*n* = 75)	Sham acupuncture + Metformin (*n* = 77)	Sham acupuncture + Placebo (*n* = 77)	
Age, years	26.97 ± 3.94	26.03 ± 4.70	26.33 ± 4.14	0.511	28.40 ± 4.42	28.76 ± 4.08	27.31 ± 3.69	0.101
BMI, kg/m^2^	21.26 ± 1.74	21.15 ± 1.92	21.26 ± 1.76	0.885	28.98 ± 3.06	29.45 ± 3.87	29.50 ± 3.87	0.652
Waist, cm	74.61 ± 6.90	75.89 ± 5.24	74.97 ± 5.85	0.659	93.21 ± 7.85	95.62 ± 8.62	94.78 ± 9.84	0.288
WHR	0.82 ± 0.05	0.84 ± 0.07	0.83 ± 0.05	0.329	0.90 ± 0.06	0.92 ± 0.06	0.90 ± 0.06	0.299
Acne score	0.38 ± 0.59	0.47 ± 0.74	0.46 ± 0.56	0.789	0.66 ± 0.93	0.66 ± 0.88	0.69 ± 0.89	0.976
Hirsutism score	3.27 ± 3.36	3.22 ± 2.99	3.51 ± 3.37	0.919	3.56 ± 3.71	4.41 ± 4.58	3.91 ± 4.24	0.506
Fasting serum levels								
HOMA-IR	3.04 ± 1.04	3.16 ± 1.21	3.32 ± 1.44	0.629	4.94 ± 2.36	5.79 ± 2.97	4.89 ± 2.16	0.067
FPG, mmol/L	5.27 ± 0.34	5.10 ± 0.40	5.09 ± 0.34	0.060	5.46 ± 0.77	5.38 ± 0.62	5.24 ± 0.58	0.158
FINS, mU/L	13.08 ± 4.82	13.86 ± 4.39	14.67 ± 5.93	0.404	20.18 ± 8.81	23.98 ± 11.23	20.90 ± 8.60	0.055
Glucose_AUC_, mmol/L × min	14.41 ± 2.47	14.74 ± 3.20	14.48 ± 3.00	0.881	16.50 ± 3.05	16.19 ± 3.50	15.39 ± 3.38	0.144
Insulin_AUC_, mU/L × min	190.10 ± 138.13	207.69 ± 108.53	235.24 ± 150.35	0.346	279.38 ± 133.90	302.11 ± 168.21	273.92 ± 150.45	0.518
HOMA-β, %	157.05 ± 89.62	185.37 ± 75.41	193.47 ± 84.00	0.148	222.99 ± 106.75	279.19 ± 158.95	268.18 ± 167.23	0.076
C-peptide, nmol/L	0.76 ± 0.17	0.84 ± 0.35	0.81 ± 0.31	0.424	1.15 ± 0.65	1.15 ± 0.40	1.08 ± 0.32	0.596
HbA1C, %	5.11 ± 0.33	5.19 ± 0.44	5.24 ± 0.33	0.275	5.44 ± 0.36	5.43 ± 0.41	5.42 ± 0.35	0.985
TC, mmol/L	4.80 ± 0.74	4.75 ± 0.78	4.96 ± 0.90	0.522	4.89 ± 0.92	5.51 ± 5.69	4.66 ± 0.84	0.339
TG, mmol/L	1.06 ± 0.52	1.11 ± 0.55	1.10 ± 0.50	0.886	1.85 ± 1.04	1.96 ± 1.42	1.57 ± 1.00	0.146
LDL-C, mmol/L	2.83 ± 0.49	2.87 ± 0.60	2.99 ± 0.66	0.476	3.19 ± 0.70	3.27 ± 0.81	3.03 ± 0.79	0.196
HDL-C, mmol/L	1.52 ± 0.35	1.46 ± 0.30	1.45 ± 0.32	0.609	1.20 ± 0.21	1.16 ± 0.21	1.23 ± 0.22	0.263
ApoA-1, mmol/L	1.32 ± 0.20	1.27 ± 0.14	1.30 ± 0.18	0.442	1.18 ± 0.17	1.14 ± 0.21	1.18 ± 0.19	0.324
Apo B, mmol/L	0.81 ± 0.17	0.81 ± 0.18	0.87 ± 0.20	0.293	0.96 ± 0.21	0.99 ± 0.24	0.90 ± 0.22	0.051
LH, IU/L	11.31 ± 5.84	12.52 ± 6.30	12.80 ± 7.26	0.588	9.42 ± 6.71	9.63 ± 4.91	9.79 ± 6.57	0.942
FSH, IU/L2	5.76 ± 1.84	5.76 ± 1.63	6.28 ± 1.32	0.283	5.88 ± 1.87	5.94 ± 1.65	5.86 ± 1.58	0.963
LH/FSH, IU/L	2.12 ± 1.11	2.26 ± 1.17	2.04 ± 1.03	0.689	1.66 ± 0.85	1.67 ± 0.82	1.64 ± 0.75	0.975
Total T, nmol/L	2.35 ± 1.08	2.37 ± 0.80	2.70 ± 1.03	0.228	2.07 ± 0.74	2.13 ± 1.30	2.04 ± 0.76	0.886
SHBG, nmol/L	47.06 ± 20.95	42.17 ± 17.27	44.02 ± 22.25	0.587	26.26 ± 13.38	27.73 ± 20.80	29.17 ± 15.94	0.633
FAI	5.89 ± 3.80	6.68 ± 3.40	7.46 ± 4.07	0.216	10.46 ± 7.28	10.74 ± 8.71	9.19 ± 6.01	0.514

BMI, body mass index; WHR, waist-to-hip ratio; HOMA-IR, homeostatic model of assessment for insulin resistance; FPG, fasting plasma glucose; FINS, fasting insulin; Glucose_AUC_, the area under the curve during the oral glucose tolerance test (OGTT) for glucose (using the trapezoidal rule); Insulin_AUC_, the area under the curve during the OGTT for insulin (using the trapezoidal rule); HOMA-β, homeostatic model assessment for beta cell function; HbA1C, hemoglobin A1c; TC, total cholesterol; TG, triglycerides; LDL-C, low-density lipoprotein cholesterol; HDL-C, high-density lipoprotein cholesterol; ApoA-1, apolipoprotein A-1; Apo B, apolipoprotein B; LH/FSH, LH to FSH ratio; Total T, total testosterone; SHBG sex hormone binding globulin; FAI, free androgen index.

### Primary outcome

For the overweight/obese women, true acupuncture was found to be less effective than metformin in improving HOMA-IR levels at 4 months after the baseline visit (Table 3, *p* = 0.004) but the efficacy was similar after 7 months (Table 3, *p* = 0.471). There were no significant declines in HOMA-IR levels compared to baseline at either 4 or 7 months after the baseline visit (*p* > 0.05). In contrast, for the lean women group, true acupuncture had similar efficacy to metformin and markedly decreased HOMA-IR at both 4 and 7 months after the baseline visit (*p* < 0.001). The sham acupuncture group also showed a significant decrease in HOMA-IR levels after 4 months (*p* = 0.018), but this effect was not observed after 7 months (*p* = 0.059). Compared with the overweight/obese women, the efficacy of true acupuncture was much better for the lean women after 4 months (*p* = 0.021). Interestingly, the efficacy of true acupuncture between overweight/obese and lean women was similar after 7 months (*p* = 0.110). Moreover, in the multivariate analysis of HOMA-IR improvement after 4 months of true acupuncture treatment, overweight/obese (OR: 0.217, 95% CI:0.052–0.900, *p* = 0.035), FAI (OR:0.840, 95% CI:0.714–0.988, *p* = 0.035), and HDL-C (OR:35.138, 95% CI:2.249–548.887, *p* = 0.011, Table 4) independently increased the risk of HOMA-IR improving; FAI (OR:1.161, CI:1.018–1.326, *p* = 0.026) and FPG (OR:3.203, CI:1.171–8.759, *p* = 0.023) independently increased the risk of HOMA-IR improving after 4 months of metformin treatment (Table 5).

**Table 3:** Changes in HOMA-IR between groups.

		True acupuncture + Placebo	Sham acupuncture + Metformin	Sham acupuncture + Placebo	*p*-value
Lean	Follow-up at 4 months after baseline visit	35	31	33	
	HOMA-IR at 4 months after baseline visit	2.27 ± 1.09	2.41 ± 1.30	2.72 ± 1.23	0.301
	Follow-up at 7 months after baseline visit	33	28	28	
	HOMA-IR at 7 months after baseline visit	2.32 ± 0.93	2.52 ± 0.99	2.54 ± 1.22	0.652
	^m^Δ	−0.73 ± 1.08	−0.64 ± 1.37	−0.54 ± 1.26	0.818
	^y^Δ	−0.72 ± 0.81	−0.54 ± 1.06	−0.71 ± 1.91	0.845
	^m^*p*-value	<0.001	0.014	0.018	
	^y^*p*-value	<0.001	0.012	0.059	
Overweight/obese	Follow-up at 4 months after baseline visit	65	65	62	
	HOMA-IR at 4 months after baseline visit	4.56 ± 2.85	4.62 ± 2.40	5.01 ± 2.94	0.599
	Follow-up at 7 months after baseline visit	61	54	58	
	HOMA-IR at 7 months after baseline visit	5.05 ± 6.54	5.01 ± 3.07	4.57 ± 2.33	0.808
	^m^Δ	−0.03 ± 1.99	−0.98 ± 2.08	0.18 ± 2.29	0.004^†^
	^y^Δ	0.46 ± 5.58	−0.26 ± 2.51	−0.03 ± 3.85	0.471
	^m^*p*-value	0.992	<0.001	0.519	
	^y^*p*-value	0.524	0.442	0.310	
	^a^*p*-value	0.021	0.347	0.105	
	^b^*p*-value	0.110	0.485	0.419	

^m^Δ Change from baseline to 4 months after the baseline visit. ^y^Δ Changes from baseline to 7 months after the baseline visit. ^m^*p*-value: baseline versus 4 months after the baseline visit. ^y^*p*-value: baseline versus 7 months after the baseline visit. ^a^*p*-value: comparison of ^m^Δ between the lean and overweight/obese groups. ^b^*p*-value: comparison of ^y^Δ between the lean and overweight/obese groups. ^†^True acupuncture + Placebo vs Sham acupuncture + Metformin.

**Table 4:** Univariate and multivariate logistic regression for HOMA-IR improvement in 100 patients after 4 months of true acupuncture treatment.

	Univariate analysis			Multivariate analysis		
	OR	95%CI	*p*-value	OR	95%CI	*p*-value
Overweight/obese	0.284	0.108–0.748	0.011	0.217	0.052–0.900	0.035
FAI	0.871	0.792–0.958	0.005	0.840	0.714–0.988	0.035
SHBG	1.040	1.011–1.070	0.006	0.999	0.949–1.051	0.961
FPG	2.996	1.057–8.486	0.039	4.075	0.825–20.120	0.085
HDL-C	8.178	1.608–41.589	0.011	35.138	2.249–548.887	0.011

HOMA-IR, homeostatic model of assessment for insulin resistance; FAI, free androgen index; SHBG, sex hormone binding globulin; FPG, fasting plasma glucose; HDL-C, high-density lipoprotein cholesterol; OR, odds ratio; CI, confidence interval.

**Table 5:** Univariate and multivariate logistic regression for HOMA-IR improvement in 96 patients after 4 months of metformin treatment.

	Univariate analysis			Multivariate analysis		
	OR	95%CI	*p*-value	OR	95%CI	*p*-value
Overweight/obese	0.800	0.315–2.027	0.637	0.668	0.201–2.224	0.511
FAI	1.071	0.984–1.166	0.111	1.161	1.018–1.325	0.026
SHBG	1.008	0.985–1.032	0.504	1.021	0.977–1.067	0.352
FPG	2.297	0.942–5.604	0.068	3.203	1.171–8.759	0.023
HDL-C	1.816	0.431–7.660	0.416	1.767	0.269–11.614	0.553

HOMA-IR, homeostatic model of assessment for insulin resistance; FAI, free androgen index; SHBG sex hormone binding globulin; FPG, fasting plasma glucose; HDL-C, high-density lipoprotein cholesterol; OR, odds ratio; CI, confidence interval.

### Secondary outcomes

At the 4-month follow-up visit, the overweight/obese women who received true acupuncture treatment showed a significant improvement in glucose_AUC_ compared to those who received metformin or sham acupuncture (Table 6, *p* = 0.019). Metformin was found to be more effective than true acupuncture in decreasing BMI, FPG, and FAI, as well as improving HOMA-β (Table 6, *p* < 0.05). No significant differences were observed among lean women (Table 6, *p* > 0.05).

**Table 6:** The changes in secondary outcomes at 4 months after the baseline visit.

	True acupuncture + Placebo	Sham acupuncture + Metformin	Sham acupuncture + Placebo	*p*-value
Lean				
BMI	−0.56 ± 0.76	−0.63 ± 1.27	−0.54 ± 0.67	0.931
No. of subjects	35	31	33	
WHR	0.00 ± 0.35	−0.01 ± 0.07	−0.01 ± 0.04	0.416
No. of subjects	35	31	33	
FPG	−0.32 ± 0.44	−0.21 ± 0.56	−0.09 ± 0.38	0.268
No. of subjects	35	31	33	
FINS	−3.37 ± 5.62	−4.28 ± 7.50	−3.80 ± 6.80	0.849
No. of subjects	35	31	33	
Glucose_AUC_	−0.58 ± 2.79	0.75 ± 2.61	−0.87 ± 3.02	0.054
No. of subjects	35	31	33	
Insulin_AUC_	−38.15(−151.43 to 75.13)	−39.58 (−128.97 to 49.81)	−13.71 (−178.00 to 150.58)	0.651
No. of subjects	34	31	33	
HOMA-β	−8.37 (−100.26 to 83.52)	32.35 (−252.48 to 317.18)	−26.53 (−113.78 to 60.72)	0.395
No. of subjects	35	31	33	
C-peptide	−0.09 ± 0.19	−0.10 ± 0.38	−0.07 ± 0.25	0.889
No. of subjects	34	31	33	
HbA1C	0.04 ± 0.30	0.01 ± 0.30	0.01 ± 0.21	0.884
No. of subjects	35	31	33	
Total T	0.14 ± 0.84	−0.02 ± 0.68	0.07 ± 0.82	0.713
No. of subjects	35	31	32	
FAI	−0.31 ± 2.02	−1.02 ± 2.64	−0.66 ± 2.47	0.487
No. of subjects	34	30	32	
Acne score	0.00 ± 0.77	0.06 ± 0.81	0.00 ± 0.71	0.927
No. of subjects	35	31	33	
Hirsutism score	−0.15 ± 0.86	−0.23 ± 0.90	−0.03 ± 0.39	0.558
No. of subjects	35	31	33	
Overweight/obese				
BMI	−0.75 ± 1.33	−1.36 ± 1.38	−0.58 ± 1.15	0.002^†‡^
No. of subjects	61	65	65	
WHR	−0.01 ± 0.05	−0.01 ± 0.04	−0.01 ± 0.05	0.764
No. of subjects	61	65	65	
FPG	−0.16 ± 0.83	−0.06 ± 0.52	0.06 ± 0.66	0.177
No. of subjects	62	65	65	
FINS	0.57 ± 7.41	−3.98 ± 7.38	0.01 ± 8.43	0.002^†‡^
No. of subjects	62	65	65	
Glucose_AUC_	−1.20 ± 2.59	−0.08 ± 1.98	−0.20 ± 2.65	0.019^†§^
No. of subjects	62	64	63	
Insulin_AUC_	−51.49 (−153.27 to 50.29)	−58.92 (−188.98 to 71.14)	−22.03 (−161.75 to 117.72)	0.214
No. of subjects	61	64	65	
HOMA-β	29.99 (−100.03 to 160.01)	−48.65 (−159.93 to 62.63)	2.04 (−182.09 to 186.17)	0.009^†‡^
No. of subjects	62	65	65	
C-peptide	−1.28 ± 0.59	−0.06 ± 0.30	−0.01 ± 0.28	0.260
No. of subjects	62	65	64	
HbA1C	−0.05 ± 0.27	−0.08 ± 0.30	−0.01 ± 0.23	0.356
No. of subjects	62	64	63	
Total T	0.05 ± 0.76	−0.21 ± 1.00	−0.02 ± 0.61	0.164
No. of subjects	60	62	60	
FAI	0.14 ± 3.97	−2.00 ± 6.02	−0.30 ± 4.35	0.047^†^
No. of subjects	59	61	57	
Acne score	−0.23 ± 0.74	−0.20 ± 0.96	−0.26 ± 0.76	0.905
No. of subjects	62	66	65	
Hirsutism score	−0.18 ± 0.86	−0.02 ± 0.33	−0.18 ± 0.85	0.315
No. of subjects	62	66	65	

FPG, fasting plasma glucose; FINS, fasting insulin; Glucose_AUC_, the area under the curve during the oral glucose tolerance test (OGTT) for glucose (using the trapezoidal rule); Insulin_AUC_, the area under the curve during the OGTT for insulin (using the trapezoidal rule); HOMA-β, homeostatic model assessment for beta cell function; HbA1C, hemoglobin A1c; Total T, total testosterone; FAI, free androgen index. ^†^True acupuncture + Placebo vs Sham acupuncture + Metformin. ^‡^Sham acupuncture + Metformin vs Sham acupuncture + Placebo. ^§^True acupuncture + Placebo vs Sham acupuncture + Placebo.

## Discussion

A *post hoc* analysis of the effect of acupuncture and metformin on insulin sensitivity in women with PCOS and IR across different BMI subgroups was performed. As a result of the present study, the following main findings were found. (1) Overweight/obese women who participated in the study were generally older and had higher levels of Waist, WHR, Acne score, baseline HOMA-IR, FPG, FINS, Glucose_AUC_, Insulin_AUC_, HOMA-β, C-peptide, HbA1C, TG, LDL-C, and FAI, but had lower levels of baseline HDL-C, ApoA-1, Apo B, LH, FSH, LH/FSH, Total T, and SHBG. (2) True acupuncture was less effective than metformin in improving HOMA-IR at 4 months after the baseline visit and the efficacy was similar at 7 months after the baseline visit for overweight/obese women with PCOS and IR. (3) HOMA-IR levels did not markedly decline either at 4 or 7 months after the baseline visit after true acupuncture treatment for overweight/obese women with PCOS and IR. (4) For the lean women group, true acupuncture had similar efficacy to metformin and markedly decreased HOMA-IR at both 4 and 7 months after the baseline visit. (5) Compared with overweight/obese women with PCOS and IR, the efficacy of true acupuncture was much better for lean women at 4 months after baseline visit. (6) The efficacy of true acupuncture between overweight/obese and lean women with PCOS and IR was similar 7 months after the baseline visit. (7) Being overweight/obese, FAI, and HDL-C levels independently increased the risk of HOMA-IR improving at 4 months after the baseline visit. (8) True acupuncture significantly improved the glucose_AUC_ compared with metformin or sham acupuncture, and metformin was superior to true acupuncture in decreasing BMI, FPG, and FAI and improving HOMA-β for the overweight/obese women with PCOS and IR, however, no significant differences were observed among the lean women. The *post hoc* analysis study demonstrated the differences in acupuncture and metformin in improving insulin sensitivity in women with PCOS IR between the overweight/obese and lean groups.

PCOS is a common clinical disease often associated with IR. It is widely believed that acupuncture can reduce obesity and type 2 diabetes by increasing insulin sensitivity (13). After acupuncture treatment, prospective studies showed significant improvements in HOMA-IR in women with PCOS and IR (18, 19). The original study found that true acupuncture decreased HOMA-IR in line with previous non-randomized studies, but it was not superior to metformin or sham acupuncture (14). However, there is little data on the differences between overweight/obese and lean patients with PCOS and IR after acupuncture treatment. Moreover, there is no study comparing the efficacy of acupuncture and metformin treatment in patients with PCOS and IR between overweight/obese and lean groups. Therefore, this *post hoc* analysis aimed to investigate the effectiveness of acupuncture and metformin in improving insulin sensitivity between overweight/obese and lean patients. In the *post hoc* analysis of the randomized controlled trial, it was found that true acupuncture had similar efficacy to metformin and significantly reduced HOMA-IR levels for lean women, regardless of whether they were assessed at 4 or 7 months after the baseline visit. Interestingly, the sham acupuncture group also experienced a significant decline in HOMA-IR levels after 4 months, which is not surprising given that sham acupuncture is not an inert procedure. As a result, the expected difference between true and sham acupuncture after 4 months of treatment was 20%. However, the efficacy of true acupuncture was not obvious for the overweight/obese women group. In addition, true acupuncture improved glucose metabolism by reducing glucose_AUC_ for the overweight/obese women group when compared to metformin which was in line with the original study (14). The improved glucose metabolism observed with acupuncture is important because this might reduce the risk of type 2 diabetes. However, the efficacy was not observed in the lean women group. All of this information suggested that acupuncture can be used as an auxiliary treatment for PCOS patients with IR and BMI management is meaningful for PCOS patients with IR to achieve better acupuncture efficacy. This association may be important for clinicians to consider in the context of acupuncture treatment.

PCOS is a prevalent condition that affects women of reproductive age and has various reproductive, metabolic, and psychological implications and lean women present with IR in a form that is mechanistically different from IR caused by overweight and obesity, which further exacerbate IR5. Adiponectin and leptin are two factors that have been extensively studied in relation to overweight/obese women. Wang et al. (20) suggested that low levels of serum adiponectin and high levels of serum resistin may have significant roles in the development of insulin resistance in PCOS patients. A previous study found that lean women with PCOS had higher levels of serum leptin, whereas this was not the case for overweight/obese women. Adiponectin demonstrated a negative correlation with insulin resistance, BMI, total testosterone, triglyceride, and LDL levels. In contrast, leptin showed an opposite correlation pattern, reversing the trends noted with adiponectin in relation to insulin resistance, BMI, total testosterone, triglyceride, and LDL levels. Additionally, a negative association was observed between adiponectin and leptin levels (21). Therefore, overweight/obesity may have an important impact on metabolic complications in women with PCOS. When studying the efficacy of treatment in women with PCOS, it is crucial to consider overweight/obese and lean women separately. According to international evidence-based guidelines for PCOS, managing BMI is considered the primary treatment approach. In the multivariate logistic regression for HOMA-IR improvement after 4 months of acupuncture treatment, being overweight/obese was an independent risk factor. These data demonstrate that overweight/obesity may affect the therapeutic effect of acupuncture. While further research is required to validate whether being overweight or obese is an independent risk factor for improved insulin sensitivity after acupuncture treatment, this possibility can be explained through plausible mechanisms. Firstly, certain studies have demonstrated a correlation between decreased insulin sensitivity and lower levels of HDL-C (22, 23), which corresponded with our outcome in the logistic regression analysis. Therefore, it may be the reason that overweight/obese patients with lower levels of HDL-C do not achieve similar efficacy to lean patients. Furthermore, a probable theory suggests that PCOS is associated with hyperandrogenism and overweight/obese patients, with high levels of FAI, may aggravate IR by increasing circulating irisin (24) and we did not observe an obvious improvement in FAI after acupuncture treatment among overweight/obese patients. Another potential reason for the development of IR in patients with PCOS is the role of vitamin D, independent of BMI. It has been suggested that vitamin D signaling through the vitamin D receptor (VDR) improves the expression of insulin receptor mRNA, leading to enhanced insulin production and release (25). Additionally, it has been found that vitamin D can directly inhibit certain pro-inflammatory cytokines that contribute to the development of IR. Notably, there is evidence indicating that vitamin D deficiency is common among obese individuals with PCOS (26). Further studies are needed to confirm this.

This study undoubtedly has some limitations. First, when considering the outcomes for overweight/obese patients, it should be noted that these were exploratory *post hoc* subgroup analyses of the original trial that were not prespecified in the study designs, therefore, the result needs to be treated with caution. Second, the number of patients was relatively small and may have resulted in statistical bias. In China, women with PCOS are typically treated with personalized acupuncture and moxibustion, following the principles of Chinese medicine. However, in this study, a fixed acupuncture protocol was used. It is worth considering that a personalized protocol, similar to the approach used in treating other medical conditions, may yield better results. Therefore, future research should focus on conducting additional studies with longer follow-up periods.

## Conclusion

The present study demonstrates that for overweight/obese women with PCOS and IR, acupuncture appeared to be less effective than metformin in improving insulin sensitivity but acupuncture shared a similar efficacy with metformin for lean women. Therefore, overweight/obesity may affect the effectiveness of acupuncture treatment. Further dedicated studies are needed to confirm these findings.

## Data availability statement

The original contributions presented in the study are included in the article/supplementary material, further inquiries can be directed to the corresponding authors.

## Ethics statement

The studies involving human participants were reviewed and approved by the Institutional Review Board of the First Hospital of Guangzhou Medical University. The studies were conducted in accordance with the local legislation and institutional requirements. The participants provided their written informed consent to participate in this study.

## Author contributions

ZW and DS: concept and design, critical revision of the manuscript for important intellectual content, and study supervision. JC and ZD: drafting of the manuscript. GN: statistical analysis. All authors: acquisition, analysis and interpretation of data. All authors read and approved the final manuscript.

## Footnotes

^1^ https://www.stata.com/stata14/
